# The Evolving Landscape of the Economics of HIV Treatment and Prevention

**DOI:** 10.1371/journal.pmed.1001174

**Published:** 2012-02-14

**Authors:** Bohdan Nosyk, Julio S. G. Montaner

**Affiliations:** 1 British Columbia Centre for Excellence in HIV/AIDS, St. Paul's Hospital, Providence Health Care, Vancouver, British Columbia, Canada; 2 Division of AIDS, Department of Medicine, University of British Columbia, Vancouver, British Columbia, Canada

## Abstract

Bohdan Nosyk and Julio Montaner argue that the cost-effectiveness of HAART roll out has been significantly underestimated because economic analyses haven't yet taken into account the beneficial impact of HAART on HIV transmission.

**Summary Points**

- In 2010 for the first time there was a plateau in the growth of the AIDS epidemic, but the current global financial crisis and drop in AIDS funding are threatening the sustainability of this achievement.
- Growing evidence suggests that HAART is not just life-saving for those with HIV, but also a highly effective means of preventing HIV transmission. Despite this evidence, there is lingering ambivalence about the expansion of HAART coverage.
- In this Essay we argue that the cost-effectiveness of HAART roll out has been significantly underestimated, as economic analyses have thus far not considered the secondary benefits of HAART, chief among them the impact of HAART on HIV transmission.
- We argue that the strategic value of expanded HIV testing and expansion of HAART coverage has dramatically increased. This has opened the door for the possibility of wide-scale implementation of “Seek, Test, Treat and Retain” programs as a means to control HIV- and AIDS-related morbidity, mortality, and transmission at once.

## Highly Active Antiretroviral Therapy: Treatment as Prevention of HIV/AIDS

The global AIDS pandemic has taken a major toll worldwide whether it is in terms of human suffering, lives lost, or economic impact. At the end of 2009, an estimated 33.3 million people were living with HIV/AIDS, with a total of 24 million accumulated AIDS-related deaths worldwide and 2.6 million new infections in 2009 alone [1].

For the first time, in 2010, there has been a plateau in the growth of the epidemic [2]. However, the current protracted global financial crisis is threatening the sustainability of this achievement, as the Joint United Nations Programme on HIV/AIDS (UNAIDS) reported a 10% drop in funding from 2009 to 2010 as United States and European governments reduce funding commitments [2]. Meanwhile, an estimated $US22–23 billion annually will be needed to fight the HIV pandemic by 2015, over 40% more than the US$16 billion allocated in 2010 [3]. This has become particularly worrisome, as accumulating evidence suggests that the use of highly active antiretroviral therapy (HAART) is not just a life-saving strategy for those infected with HIV, but it is also a highly effective means of preventing HIV transmission.

The preventive value of HAART has now been definitively proven in the context of serodiscordant couples, where the immediate use of HAART by the partner with HIV has led to a relative reduction of 96% in the number of linked HIV-1 transmissions, as compared with delayed therapy in a landmark randomized trial [4]. While this result was found solely in serodiscordant heterosexual couples, the basic mechanism of HAART reducing HIV-1-RNA to undetectable levels in blood, genital secretions, and breast milk applies to all modes of transmission. As such, similar protective benefits have been reported among injection drug users in Vancouver, British Columbia, Canada [5], and Baltimore, Maryland, US [6], as well as population-based studies [7],[8].

## Current Evidence on the Cost-Effectiveness of HAART and HIV Testing and Prevention Strategies

HAART has long been deemed to be a cost-effective intervention, as it is effective in reducing morbidity and mortality and increasing life-years and quality-adjusted life years (QALYs) gained [9]. However, HAART is associated with substantial up-front costs, and therefore, there has been some lingering ambivalence regarding the expansion of HAART coverage [2]. Indeed, waiting lists to access HAART continue to be a recurrent theme in North America and in resource-limited settings [10],[11]. This is to a large extent based on an incomplete assessment of the true cost-effectiveness of HAART roll out, as illustrated by a recent systematic review. While 89.7% of studies deemed HAART to be cost-effective, only 54.2% of the counseling, testing, and referral strategies evaluated were found to be cost-effective by commonly used thresholds in economic evaluation [12]. Notably, the majority of these studies focused solely on the patient-centered benefits of HAART use. As such, they did not consider the now abundantly documented secondary benefits of HAART, chief among them the impact of HAART on HIV transmission. The additional return on the investment generated by decreases in HIV transmission associated with the use of HAART markedly enhances the cost-effectiveness of the overall expanded roll out of HAART strategy [13]. Consequently, it is likely that the overall cost-effectiveness of HAART roll out was significantly underestimated in the majority of these studies [4]–[8]. The evidence is clear: treatment prevents morbidity, mortality, and transmission. From this point on, we argue that these three endpoints should be considered together when evaluating the cost-effectiveness of HAART.

## Moving Forward

As we move forward, additional endpoints will need to be captured to fully understand the economic impact of HAART expansion. For instance, HAART is now accepted as a highly effective means to prevent tuberculosis [14]. Expanded treatment will also prevent orphanhood, which also carries a substantial economic burden. One study found that the annual costs of childcare in Rwanda and Malawi were equal to or greater than the annual per capita GDP in those countries [15]. Finally, HAART will also have a positive impact on productivity, particularly in Africa, where AIDS was conservatively estimated to have reduced GDP growth rates by 2%–4% per year in one study [16].

While challenges in implementation of early detection programs exist [17], particularly in light of the high risk of transmission in acute and early HIV infection [18], the impact of HAART on HIV transmission has nonetheless profoundly shifted the cost-effectiveness of this intervention. As a result, the strategic value of expanded HIV testing and expansion of HAART coverage has dramatically increased. We believe this should open the door for wide-scale implementation of “Seek, Test, Treat and Retain” [19] programs as a means to control HIV and AIDS-related morbidity, mortality, and transmission at once.

## Abbreviations

AIDS: acquired immune deficiency syndrome
HAART: highly active antiretroviral therapy
HIV: human immunodeficiency virus
QALYs: quality-adjusted life years
UNAIDS: Joint United Nations Programme on HIV/AIDS

## References

[1] UNAIDS (2010). UNAIDS report on the global AIDS epidemic 2010.. http://www.unaids.org/globalreport/global_report.htm.

[2] Lomborg B, Piot P (27 September 2011). Re-thinking the fight against AIDS.. http://online.wsj.com/article/SB10001424053111904194604576583071258290228.html?KEYWORDS=HIV.

[3] Fauci AS (2011). AIDS: Let science inform policy.. Science.

[4] Cohen MS, Chen YQ, McCauley M, Gamble T, Hosseinipour MC (2011). Prevention of HIV-1 infection with early antiretroviral therapy.. N Engl J Med.

[5] Wood E, Kerr T, Marshall BD, Li K, Zhang R (2009). Longitudinal community plasma HIV-1-RNA concentrations and incidence of HIV-1 among injecting drug users: a prospective cohort study.. BMJ.

[6] Kirk G, Galai N, Astemborski J, Linas B, Celentano D (2011). Decline in community viral load strongly associated with declining HIV incidence among IDU..

[7] Montaner JSG, Lima VD, Barrios R, Yip B, Wood E (2010). Association of highly active antiretroviral therapy coverage, population viral load, and yearly new HIV diagnoses in British Columbia, Canada: A population-based study.. Lancet.

[8] Das M, Chu PL, Santos GM, Scheer S, Vittinghoff E (2010). Decreases in community viral load are accompanied by reductions in new HIV infections in San Francisco.. PLoS ONE.

[9] Walensky RP, Freedberg KA, Weinstein MC, Paltiel AD (2007). Cost-effectiveness of HIV testing and treatment in the United States.. Clin Infect Dis.

[10] Walensky RP, Paltiel AD, Freedberg KA (2002). AIDS drug assistance programs: highlighting inequalities in human immunodeficiency virus-infection health care in the United States.. Clin Infect Dis.

[11] Duff P, Kipp W, Wild TC, Rubaale T, Okech-Ojony J (2010). Barriers to accessing highly active antiretroviral therapy by HIV-positive women attending an antenatal clinic in a regional hospital in western Uganda.. J Int AIDS Soc.

[12] Hornberger J, Holodniy M, Robertus K, Winnike M, Gibson E (2007). A systematic review of cost-utility analyses in HIV/AIDS: Implications for public policy.. Med Decis Making.

[13] Johnston KM, Levy AR, Lima VD, Hogg RS, Tyndall MW (2010). Expanding access to HAART: a cost-effective approach for treating and preventing HIV.. AIDS.

[14] Badri M, Wilson D, Wood R (2002). Effect of highly active antiretroviral therapy on incidence of tuberculosis in South Africa: a cohort study.. Lancet.

[15] Bhagarva A, Bigombe B (2003). Public policies and the orphans of AIDS in Africa.. BMJ.

[16] Dixon S, McDonald S, Roberts J (2002). The impact of HIV and AIDS on Africa's economic development.. BMJ.

[17] Walensky RP, Paltiel AD, Losina E, Morris B, Scott AS (2010). Test and Treat DC: forecasting the impact of a comprehensive HIV strategy in Washington, DC.. Clin Infect Dis.

[18] Miller WC, Rosenberg NE, Rutstein SE, Powers KA (2010). Role of acute and early HIV infection in the sexual transmission of HIV.. Curr Opin HIV AIDS.

[19] Volkow N, Montaner J (2010). Enhanced HIV testing, treatment, and support for HIV-infected substance users.. JAMA.

